# Whole exome sequencing of an asbestos-induced wild-type murine model of malignant mesothelioma

**DOI:** 10.1186/s12885-017-3382-6

**Published:** 2017-06-02

**Authors:** Sophie Sneddon, Ann-Marie Patch, Ian M. Dick, Stephen Kazakoff, John V. Pearson, Nicola Waddell, Richard J. N. Allcock, Robert A. Holt, Bruce W. S. Robinson, Jenette Creaney

**Affiliations:** 1 National Centre for Asbestos Related Disease, School of Medicine and Pharmacology, QEII Medical Centre, University of Western Australia, QQ Block, 6 Verdun Street, Nedlands, WA 6009 Australia; 2 QIMR Berghofer Medical Research Institute, Brisbane, Brisbane, QLD 4006 Australia; 3 School of Pathology and Laboratory Medicine, University of Western Australia, Nedlands, WA 6009 Australia; 4 Pathwest Laboratory Medicine, Western Australia, QEII Medical Centre, Nedlands, WA 6009 Australia; 5 Michael Smith Genome Sciences Centre, BC Cancer Agency, Vancouver, BC Canada; 6 Department of Respiratory Medicine, Sir Charles Gairdner Hospital, Nedlands, WA 6009 Australia

**Keywords:** Mesothelioma, Asbestos, Exome sequencing, Cdkn2a, Mouse model, Wild-type

## Abstract

**Background:**

Malignant mesothelioma (MM) is an aggressive cancer of the pleural and peritoneal cavities caused by exposure to asbestos. Asbestos-induced mesotheliomas in wild-type mice have been used extensively as a preclinical model because they are phenotypically identical to their human counterpart. However, it is not known if the genetic lesions in these mice tumours are similar to in the human disease, a prerequisite for any new preclinical studies that target genetic abnormalities.

**Methods:**

We performed whole exome sequencing of fifteen asbestos-induced murine MM tumour cell lines from BALB/c, CBA and C57BL/6 mouse strains and compared the somatic mutations and copy number variations with those recurrently reported in human MM. We then catalogued and characterised the mutational landscape of the wild-type murine MM tumours. Quantitative RT-PCR was used to interrogate the expression of key MM genes of interest in the mRNA.

**Results:**

Consistent with human MM tumours, we identified homozygous loss of the tumour suppressor *Cdkn2a* in 14/15 tumours. One tumour retained the first exon of both of the p16INK4a and p19ARF isoforms though this tumour also contained genetic amplification of *Myc* resulting in increased expression of the c-Myc proto-oncogene in the mRNA. There were no chromosomal losses in either the *Bap1* or *Nf2* regions. One tumour harbored homozygous loss of *Trp53* in the DNA. Mutation rates were similar in tumours generated in the CBA and C57BL/6 strains when compared to human MM. Interestingly, all BALB/c tumour lines displayed high mutational loads, consistent with the known mutator phenotype of the host strain. The Wnt, MAPK and Jak-STAT signaling pathways were found to be the most commonly affected biological pathways. Mutations and copy number deletions also occurred in the Hedgehog and Hippo pathways.

**Conclusions:**

These data suggest that in the wild-type murine model asbestos causes mesotheliomas in a similar way to in human MM. This further supports the notion that the murine model of MM represents a genuine homologue of the human disease, something uncommon in cancer, and is thus a valuable tool to provide insight into MM tumour development and to aide the search for novel therapeutic strategies.

**Electronic supplementary material:**

The online version of this article (doi:10.1186/s12885-017-3382-6) contains supplementary material, which is available to authorized users.

## Background

Malignant mesothelioma (MM) is an aggressive cancer caused by DNA damage in the mesothelial cells of the pleural and peritoneal cavities that principally results following asbestos exposure [1–3]. There is a long latency between asbestos exposure and tumour development with periods of 30–50 years frequently reported. MM is almost universally fatal and median survival after diagnosis is short (9–12 months) [1, 2].

Asbestos is one of only a few carcinogens, along with cigarette smoke, UV light and several others, that can be readily identified in individuals. Importantly, asbestos induces mesotheliomas in mice which are almost identical to their human counterpart in terms of pathology, immunology and clinical behavior, which is rare in mouse modeling of cancer [4, 5]. Although this has made the mouse model a useful preclinical tool for studying mesothelioma treatments, until now there has been no detailed study of the genetic lesions associated with mouse mesothelioma to determine if such lesions also parallel the human disease. Detailed studies of the genetic lesions of human MM have only recently been published. On average, human MM tumours contain less than 1 mutation per million bases, which is lower than other malignancies associated with external carcinogens such as lung cancer and melanoma [6–8]. A variety of somatic copy number variations (CNVs) and single nucleotide variations (SNVs) have been recurrently reported in several genes. Loss of *CDKN2A*, located at 9p21, is reported in more than 70% of cases; loss of *NF2*, located at 22q, is reported in around 40% of cases; and mutations in *BAP1*, located at 3p21.1 are reported in 40–60% of cases [9–13]. Recent large scale genomic analyses have also found recurrent, low frequency mutations in a number of additional genes including *LATS2* (22%)*, CUL1* (9%)*, TP53* (8%) and *SETD2* (8%) [7, 8, 14]. Shared somatic mutations between MM tumours are rare, however several biological pathways are often reported as being dysregulated in human MM, including the Wnt, Hedgehog, Notch, Ras, p53, MAPK, mTOR and Hippo signaling pathways [7, 15, 16].

Murine models of MM are an invaluable tool for the preclinical evaluation of disease pathogenesis and for developing novel treatment strategies [17]. Recent studies have utilized mouse xenograft models and several genetically engineered mouse models to recapitulate the common mutations seen in human MM, such as *Nf2*, *Cdkn2a* and *Bap1* knockout models [18–20]. However, such models only enable the study of MM in the context of the effect of the knocked out gene of interest. Our well established asbestos-induced wild-type murine MM model has the potential to offer useful molecular insights on the natural initiation and progression of MM in response to asbestos exposure, providing the opportunity for better understanding of pathogenesis, development of novel treatments and biomarker/signature discovery. Detailed characterisation of the genomic lesions underpinning the wild-type murine MM model have, as in other cancers, lagged behind the relevant human studies. This model has been extensively characterised at the phenotypic and morphological level on the BALB/c background [5]. Gene expression has previously been characterised in the C57BL/6 strain [21] and array-comparative genomic hybridisation (aCGH) studies have identified lesions in FVB/N mice [22] however little is known about the mutational landscape of these wild-type tumours. We therefore undertook to characterize the somatic DNA lesions that underlie murine MM and characterise the mutational landscape using whole exome sequencing of fifteen independent murine MM tumour cell lines derived from three murine strains, comparing the mutations identified with those most often found in human MM.

## Methods

### Sample collection and DNA extraction

Fifteen murine MM cell lines were previously established from ascites generated following intra-peritoneal crocidolite asbestos injection into BALB/c (*n* = 4) [4], CBA (*n* = 5) [4] and C57BL/6 (*n* = 6) mice [23, 24] (Table 1). Established cell lines (median passage 14, range 8–31) were used in this study and all cell lines were confirmed free of *Mycoplasma* spp. infection by polymerase chain reaction. Cells were cultured as previously described [25]. Control DNA and RNA samples were obtained from current generation mice sourced from the Animal Resource Centre (Murdoch, WA, Australia) for each mouse strain in accordance with protocols approved by the University of Western Australia Animal Ethics Committee. DNA was extracted using standard isopropanol purification. RNA was extracted using an RNeasy Mini Kit (QIAGEN, Chadstone, Vic, Australia) and cDNA prepared using an OmniScript Reverse Transcription kit (QIAGEN) with oligo dT primers (Promega, Alexandria, NSW, Australia) following the manufacturer’s instructions.

**Table 1:** Establishment and growth rate information of wild-type asbestos-induced murine MM tumour cell lines

Strain	Cell line name	Year established	In vivo growth rate^a^	Passage sequenced
BALB/c	AB1	1992	fast	18
	AB12	1992	slow	11
	AB13	1992	slow	14
	AB22	1992	slow	8
CBA	AC16	1992	slow	24
	AC24	1992	Undetermined	31
	AC28	1992	slow	13
	AC29	1992	fast	31
	AC31	1992	fast	8
C57BL/6	AE3	2001	nil	11
	AE16	2001	nil	14
	AE17	2001	fast	13
	AE19	2001	slow	17
	BM109	2004	slow	14
	BM163	2005	slow	20

^a^Relative time taken for a 100mm^2^ subcutaneous tumour to form following an inoculation of 5 × 10^5^ cells in the flank of syngeneic mice. Growth rate slow > 80 days; Fast <40 days

### Library preparation and exome sequencing

Genomic DNA was fragmented using an S2 sonicator (Covaris, USA). Whole genome libraries were constructed using a NEBNext Ultra DNA Library kit (New England Biosciences, Ipswich, MA) with local modifications (specifically Ion Torrent sequencing adaptors were added) and the exome was enriched using a SureSelectXT Mouse All Exon kit (Agilent, Mulgrave, Vic, Australia) that targets 49.6 Mb of the murine genome (221,784 exons across 24,306 coding genes). Sequencing was performed using a Proton sequencer (Ion Torrent, Thermofisher Scientific) system according to manufacturer’s instructions at the Lotterywest State Biomedical Facility Genomics (Nedlands, WA, Australia). Two samples were pooled and sequenced on a P1 chip for 520 flows to an average depth of 42×, which resulted in 80% of the targeted regions being covered with at least an average depth of 13.2× (See Additional file 1).

### Data processing and variant detection

Sequence reads were trimmed using Cutadapt (version 1.9) [26], aligned to GRCh38/mm10 with BWA-MEM (version 0.7.13-r1126), duplicates marked with Picard (version 1.141, http://broadinstitute.github.io/picard/) and coordinates sorted using Samtools (version 1.3) [27]. Single nucleotide substitution variants were detected using a dual calling strategy using qSNP [28] and the Haplotype caller module of GATK [29]. Short indels of ≤50 bp, were called with the Haplotype. Initial read quality filtering for all variants detected included: a minimum of 35 bases in the CIGAR string indicated a match, 3 or fewer mismatches in the sequencing MD field, and a mapping quality greater than 10. High confidence variants were selected based on passing further variant characteristic filtering requirements and were used in all downstream analyses. These filtering requirements were: a minimum coverage of 8 reads in the control data and 12 reads in the tumour data and a maximum of 1000 reads; at least 5 variant supporting reads present where the variant was not within the first or last 5 bases; at least 4 of the 5 reads with individual start positions; the variant was identified in reads of both sequencing directions; the variant was not less than 5 base pairs from a mono-nucleotide run of 7 or more bases in length; and for somatic variants less than 3% variant evidence identified in the control sample. In addition, for indels a minimum of 4 strong variant supporting reads containing a maximum of 3 variant events (including the indel of interest and any other non-reference occurrence) were required. Indels that were located immediately adjacent to homopolymer regions of at least 3 base pairs and for which the inserted or deleted base were identical to the homopolymer base were filtered out as homopolymer-associated errors. Indels required a tumour variant allele frequency of >35% and all coding indels were manually reviewed using IGV [30, 31] in order to be considered high confidence. Variants were annotated with gene feature information and transcript or protein consequences using SnpEff (version 4.2) [32]. Genes of interest, such as *Bap1, Cdkn2a* and *Nf2* were visually inspected for any possible somatic mutation using IGV.

Mutation rate was calculated from the total number of missense, nonsense and silent SNVs plus coding indels divided by the size of the exome library design. Genes significantly mutated at a rate higher than background were identified using Genome MuSIC [33], where a convolution test *p*-value threshold cutoff of <0.05 was used to determine significance. Mutations that occurred in all cell lines of the same strain at the same position, but were absent from the matched control were excluded as these were considered to be strain-specific polymorphisms.

### Identification of copy number alterations

Somatic CNVs were identified using coverage information for each tumour exome and matching normal sample calculated by GATK. Normalized and log transformed read count ratios were then used to identify regions of amplification and deletion with ExomeCNV [34]. Segmentation was performed using DNAcopy [35] and segmented data was used as input for the Genetic Identification of Significant Targets in Cancer version 2.0 (GISTIC2.0) program [36] The murine reference genome for GISTIC2.0 was kindly provided by Steven Schumacher (Broad Institute, Cambridge, MA). A q-score of less than 0.1 and a confidence interval of 95% was used to determine the significance of regions of CNV across samples. Homozygous deletions of key genes of interest were visually inspected using IGV. To identify whether known tumour suppressor genes, genes in regions of amplification or deletion identified as being significant in the GISTIC2.0 the genes were cross-referenced with the Tumour Suppressor Gene Database 2.0 [37].

### Identification of altered pathways

Pathways of interest in MM were identified from the literature. Gene lists for both the human and murine versions of each pathway were extracted from KEGG [38]. All missense, nonsense and frameshift mutations were annotated with corresponding KEGG pathway information for each sample using DAVID Bioinformatics Resources 6.8 [39] and cross referenced with the pathway gene lists. The numbers of genes containing missense, nonsense and frameshift mutations in each pathway of interest was determined. Functional annotation clustering on KEGG pathway annotations using DAVID Bioinformatics Resources 6.8 and WebGestalt [40] was performed for all genes identified as being significantly mutated above background mutation rate and genes located in regions of significant amplification or deletion, with a Benjamini-Hochberg adjusted *p*-value threshold of <0.05 from both tools considered significant.

### Validation of genetic alterations of interest

Quantitative real-time polymerase chain reaction (RT-PCR) was used to determine mRNA expression. Primers for genes of interest were identified from the literature or designed using Primer-BLAST (NCBI) and purchased from GeneWorks (Thebarton, SA, Australia) (See Additional file 2). Quantitative RT-PCR was performed using SYBR® Green Universal PCR Master Mix (ThermoFisher Scientific) for 35 cycles of 95 °C for 30s, 60 °C for 30s and 72 °C for 30s. Differences in gene expression were described as fold-changes between tumour and strain-matched non-tumour sample in order to identify expression changes that may be induced by copy number variation. Sanger sequencing was performed as previously described [25] to confirm the presence of indels.

## Results

### *Cdkn2a* is commonly lost in murine MM tumour cell lines

**Fig. 1:**
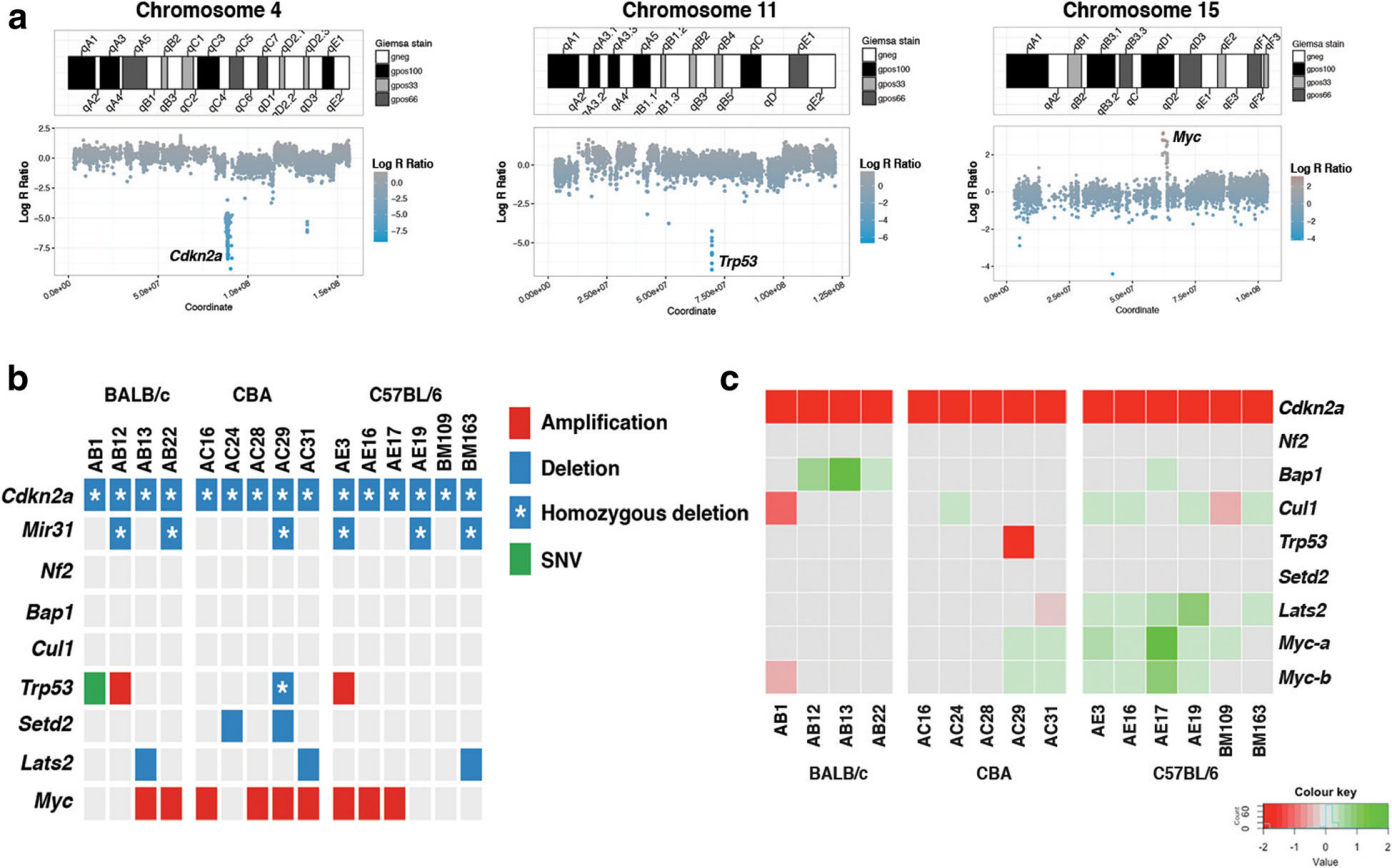
Copy number variations in genes frequently altered in human MM tumours were identified in murine MM tumour cell lines. As an example, *Cdkn2a* loss is shown in sample AB22, *Trp53* loss is shown in AC29 and *Myc* amplification is shown in AC28 (**a**). The mutation status was assessed for a group of genes selected based on frequent somatic alteration in human MM (See Additional file 3) (**b**). Scaled fold-change of the mRNA expression of each target, compared back to the strain matched control, as determined by qPCR, is shown as reduced (*red*) or increased (*green*) (**c**)

Fifteen murine MM cell lines previously established from ascites generated following intra-peritoneal crocidolite asbestos injection into BALB/c (*n* = 4), CBA (*n* = 5) and C57BL/6 (*n* = 6) mice were assessed for somatic mutations and copy number variation (CNV) aberrations (Table 1) [4, 23, 24]. Overall there were a greater number of copy number deletion events than amplifications. A total of 14 regions were significantly affected by copy number changes, including 12 regions of deletion and 2 regions of gain (See Additional file 3). The most significant region was a homozygous deletion encompassing the tumour suppressor gene *Cdkn2a* which was evident in all 15 murine MM tumour cell lines (q = 5 × 10^−17^) (Fig. 1a and b). The breakpoints and length of the region of loss varied between samples, varying from 64.2 to 4025.6 kilobases in length (Table 2). In one sample, AE17, exon 1 of both *Cdkn2a* isoforms was retained (NM_001040654.1 and NM_009877.2). Quantitative real-time polymerase chain reaction (PCR) confirmed the absence of *Cdkn2a* expression in messenger RNA in all samples (Fig. 1c).

**Table 2:** Variation in breakpoints of homozygous deletion in region of *Cdkn2a*

Sample	Range (start-end) ^a^	Length (kbp)	Co-loss of *Mtap*	Co-loss of *Mir31*
AB1	chr4:88,880,322–89,691,452	811.1	Y	N
AB12	chr4:88,603,449–91,805,387	3201.9	Y	Y
AB13	chr4:89,217,653–89,443,728	226.1	N	N
AB22	chr4:88,435,182–90,223,528	1788.3	Y	Y
AC16	chr4:89,217,686–89,307,039	89.3	N	N
AC24	chr4:88,880,305–89,688,653	808.3	Y	N
AC28	chr4:89,217,686–89,688,653	470.9	N	N
AC29	chr4:87,227,653–91,253,295	4025.6	Y	Y
AC31	chr4:89,217,686–90,223,513	1005.8	N	N
AE3	chr4:86,917,707–90,223,511	3305.8	Y	Y
AE16	chr4:89,156,673–90,223,511	1066.8	Y	N
AE17	chr4:89,217,685–89,281,894	64.2	N	N
AE19	chr4:87,784,120–90,223,511	2439.4	Y	Y
BM109	chr4:89,217,685–89,688,699	471.0	N	N
BM163	chr4:87,880,239–90,856,876	2976.6	Y	Y

^a^Loci reported as per mm10 coordinates

No other significant amplifications or deletions in genes known to be affected in human MM aside from *Cdkn2a* were detected in the dataset. However, a number of deleted loci contained known tumour suppressor genes that have been associated with other cancers, as defined by TSGene2.0, such as *Epha*, *Robo1*, *Arl6*, *Cadm2*, *Lipi*, *Prb1* and *Sir1*. The number of copy number events did not correlate with time in culture (See Additional file 1).

Copy number aberrations in candidate loci previously implicated in MM were identified in the region of *Setd2* in 2 out 15 cell lines and in *Lats2* in 3 out of 15 (20%) cell lines. However, we did not observe reduced mRNA expression of these genes (Fig. 1c) and the regions were not considered to be statistically significantly affected across the samples set through GISTIC2.0 analysis. A region on chromosome 15 encompassing the *Myc* oncogene was observed to be amplified in 9/15 samples (Fig. 1a and b). Gene expression analysis showed a 150-fold increase in expression of *Myc* in AE17 (Fig. 1c), and a range of 9-to 24-fold increased expression in the other C57BL/6 samples and two CBA samples (AC29, AC31). A homozygous deletion of *Trp53* was found in AC29 which was confirmed in the messenger RNA by the absence of product using RT-PCR.

### Somatic mutations in murine MM tumour cell lines

**Fig. 2:**
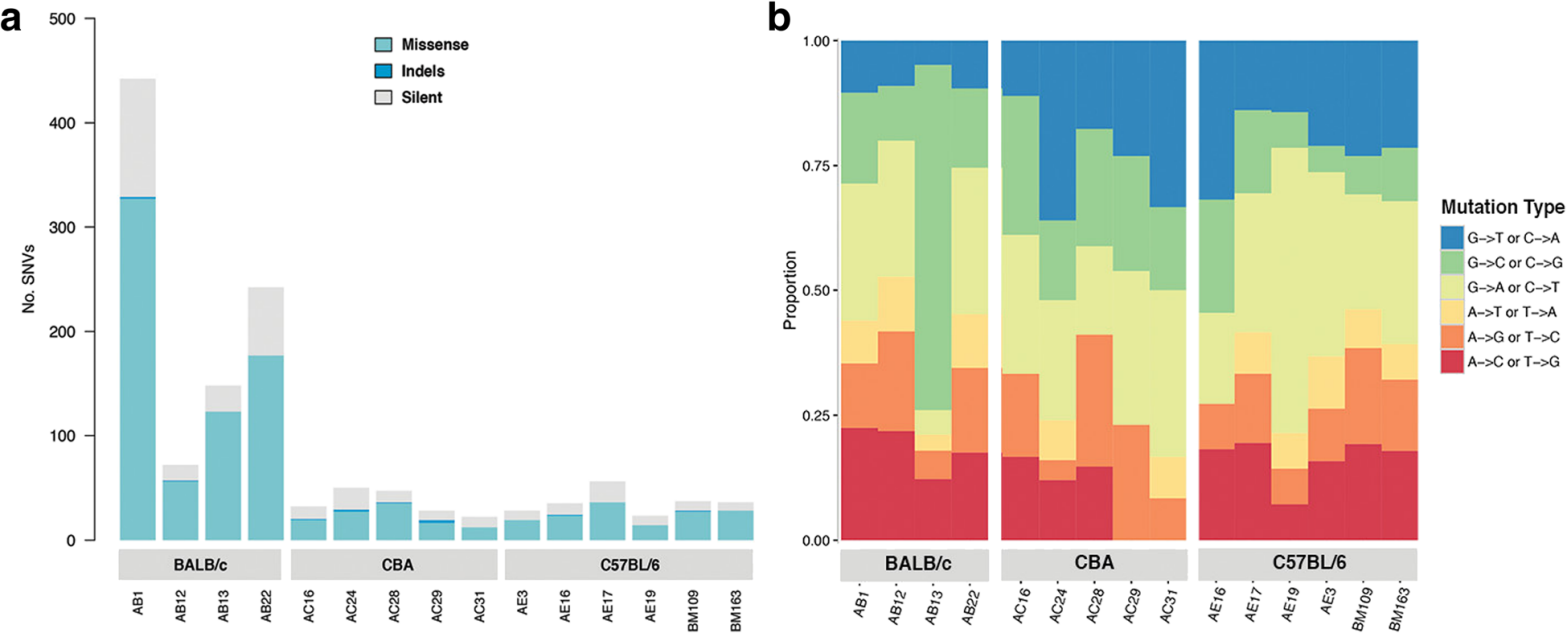
Exonic somatic mutations in murine MM cell lines showing a breakdown between missense, indels and silent mutations (**a**) and mutation type (**b**)

A total of 1286 somatic SNVs and small indels in 1136 genes were detected in the 15 samples. Of these, 899 were missense mutations, 28 were nonsense mutations, 12 were small indels and 347 were silent mutations. On average, there were 84 mutations per sample (range 22 to 438) of which 74% (average 62, range 12 to 327) were protein coding (Fig. 2a; See Additional file 1). Tumours generated in mice of the BALB/c background had a higher average number of mutations (*n* = 225) than those from the CBA (*n* = 33) and C57BL/6 (*n* = 35) strains (Fig. 2a; See Additional file 1). The median overall rate of mutation was 0.7 mutations/Mb (3.9/Mb for BALB/c, 0.6/Mb for CBA and 0.7/Mb for C57BL/6 samples). Mutation frequency among BALB/c samples was variable, ranging from 1.4–8.9 mutations/Mb (See Additional file 1).

In general, C > T and G > A mutations were highly represented in all samples (Fig. 2b). AB13 and AB22 showed marked differences to the other BALB/c samples with an abundance of C > G and A > T mutations, respectively. For the CBA samples, AC24 showed a higher proportion of G > T mutations, while the other CBA samples showed similar proportions of mutation types. Of the C57BL/6 samples, AE19 was dominated by C > T at the expense of G > A mutations (Fig. 2b).

Fifteen genes were mutated in more than one sample and were found to be significantly mutated above what would be expected as background (Table 3). Several of the genes, such as *Mmp8, Dock10, Hoxd3* and *Fat3*, have previously been reported to show mutation or altered expression in cancer and particularly metastasis [41–45]. Two genes, *Cacna2d2* and *Dbc1* are candidate tumour suppressors in other cancers [46, 47].

**Table 3:** List of genes containing >1 amino acid change causing mutation and the significance of the mutation beyond background mutation rate (convoluted *p*-value <0.05)

Gene (KEGG pathway)	Mutations	Samples affected	No. strains affected	*p*-value
Nkd1 (Wnt signalling pathway)	p.P84L|p.V47I	AB1|AB22	1 (BALB/c)	1.34E-04
Mmp8	p.L7P|p.F303 V	AB1|AB22	1 (BALB/c)	5.38E-04
Nckap5	p.P1182H, p.L1191 V, p.G768D	AB1|AB13|AE16	2 (BALB/c|C57BL/6)	9.96E-04
Dab1	p.F203C|p.L502 V	AB1	1 (BALB/c)	1.22E-03
Dsc1	p.K481 N|p.E519D	AB1|AB13	1 (BALB/c)	1.45E-03
Rftn1	p.V106G|p.W404C	AB1|AB13	1 (BALB/c)	2.43E-02
Ano4	p.N644 K|p.N406Y	AB22	1 (BALB/c)	2.07E-03
Dpp9	p.T763R|p.P403T	AB1	1 (BALB/c)	3.74E-03
Pabpc6	p.G402R|p.D45Y	AB1|AB22	1 (BALB/c)	3.29E-03
Hoxd3	p.Q7K|p.V388 M	AE17|AE19	1 (C57BL/6)	4.81E-03
Hc	p.V1332 M|p.I25M	AB22	1 (BALB/c)	3.51E-03
Dock10	p.V1546G|p.A2076V	AB1|AE17	2 (BALB/c|C57BL/6)	3.28E-03
Cacna2d2 (MAPK signalling pathway)	p.K857 M|p.R575Q	AB1|AC28	2 (BALB/c|CBA)	3.67E-03
Neurl4	p.V1163I|p.T832 K	AC29|BM163	2 (CBA|C57BL/6)	6.50E-03
Fat3	p.D2918G|p.D1300E	AB1	1 (BALB/c)	3.61E-02

There was no evidence of any mutation in genes most commonly identified as being mutated in human MM, such as *Bap1*, *Nf2* or *Lats2*. However, a single missense SNV in *Trp53* occurred in one BALB/c sample (Fig. 1a). Reduced expression of *Bap1* or *Nf2* in the messenger RNA was not observed between tumours and normal samples. However two BALB/c samples, AB12 and AB13, showed a 56- and 136-fold increase of *Bap1* in mRNA expression, respectively. The genes *Rb1* and *Pten* were intact.

### Pathway alterations in murine MM

**Fig. 3:**
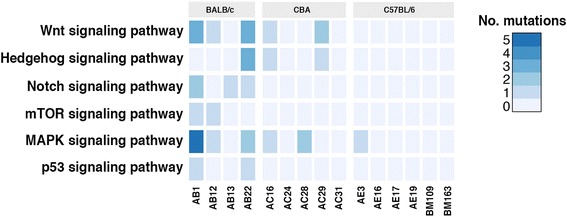
The number of missense mutations affecting pathways commonly known to be disregulated in human MM tumours. The following number of genes contained missense mutations for each pathway: 10 (Wnt signaling pathway), 5 (Hedgehog signaling pathway), 2 (Notch signaling pathway), 2 (mTOR signaling pathway), 11 (MAPK signaling pathway) and 2 (p53 signaling pathway)

Three biological pathways were enriched in the list of genes significantly mutated by somatic mutation or copy number alteration when analysed using WebGestalt and DAVID. Of note the Jak-STAT signaling pathway (WebGestalt adjusted *p*-value = 0.017; DAVID *p*-value = 0.016) were significantly altered (Table 4). Six pathways known to be dysregulated in human MM were affected by missense mutations in the murine MM tumour cell lines: 5/15 samples harbored missense mutations in genes in the Wnt signaling pathway; 3/15 in the Hedgehog signaling pathway; 3/15 in the Notch signaling pathway; 2/15 in the mTOR signaling pathway and 2/15 in the p53 signaling pathway (Fig. 3). All six pathways contained missense mutations in the BALB/c samples. The CBA samples showed mutations in the Wnt, Hedgehog and MAPK signaling pathways, while the C57BL/6 strain contained only one sample carrying a single missense mutation in the MAPK signaling pathway and no mutations in the other five pathways (Fig. 3). There were no mutations in the Ras signaling pathway, however *Lats2*, which initiates cell apoptosis through the Hippo pathway, contained copy number deletions in 3/15 samples.

**Table 4:** KEGG pathways enriched from list of significantly mutated genes and significantly amplified or deleted regions (*n* = 804)

KEGG pathway	No. mutated genes	BH adjusted *p*-value (WebGestalt)	BH adjusted *p*-value (DAVID)
Regulation of autophagy	7	1.5 × 10^−3^	5.2 × 10^−4^
RIG-I-like receptor signaling pathway	9	2.4 × 10^−3^	1.1 × 10^−2^
Jak-STAT signaling pathway	12	1.7 × 10^−2^	1.6 × 10^−2^

## Discussion

The asbestos-induced wild-type murine model of MM has the power to provide invaluable insights into the pathology of human MM, provided that the tumours are similar on a molecular basis between the species. Through whole exome sequencing of tumour cell lines developed from mesotheliomas from three murine strains, we have shown that the *Cdkn2a* gene is consistently lost in the asbestos-induced wild-type murine model of MM. This parallels the situation in more than 70% of human MM tumours [12]. Previously, in a genetically engineered MM mouse model, heterozygous for *Nf2* ^+/−^, a high rate of homozygous deletion of *Cdkn2a* was observed in MM tumours induced by asbestos exposure [19]. In the human context *CDKN2A* loss, deletion of *NF2* and/or mutation of *BAP1* has been reported in up to 80% of clinical cases [8]. In our model we did not find mutations, copy number deletions or reduced mRNA expression of *Bap1*, however, loss of BAP1 protein expression has been reported in up to 25% of cases where no genetic mutations were detected [9]. Dysregulation of the Hippo pathway is often reported in human MM, typically through mutations or copy number aberrations of *NF2*, *YAP* and/or *LATS2* [14, 48]. No mutations in *Lats2* were observed in this study, however 20% of samples in this study contained a copy number deletion of *Lats2.* With post-transcriptional modification potentially accounting for loss of these tumour suppressors in the mouse asbestos induced MM model it is not possible to conclusively say that *Cdkn2a* deletion is an independent driver of MM. However it seems that homozygous loss of the *Cdkn2a* gene is sufficient for the development of asbestos-induced MM in the absence of other common MM driver mutations at the genetic and transcriptomic level in this model.

Though most human MMs develop in the pleura, the murine model used in this study developed in the peritoneum. Despite this difference, the developmental characteristics of murine MM faithfully mimic human MM [4]. Pleural and peritoneal MM are different in terms of prognosis and treatment options, however the molecular features of peritoneal MM are not as well characterised as pleural MM, given the rarity of the diagnosis. It is not uncommon to have secondary pleural invasion from peritoneal MM and vice versa, and in these cases the distinction between the two types is clinically difficult [49]. The histological feature of pleural and peritoneal MM are generally identical, and on this basis, the molecular characterisation of murine MM in this study will provide the necessary basis for future studies seeking to target specific molecular pathways for therapy.

The asbestos-exposed wild-type murine model of MM displayed a high proportion of chromosomal loss compared to gain, which is a characteristic feature of human MM. Human MM tumours display recurrent losses in 1p21-p22, 9p21-p22, 3p21, 6q15-q21, 17p13.1 and 22q12.2 [50, 51]. Taking into account the locations of *BAP1*, *NF2* and *CDKN2A* in the human genome, the only orthologous region containing CNV aberration in this model was the region containing *Cdkn2a* (4qC4). Previously we have shown using our MexTAg transgenic mouse model [24] that the presence of exogenous SV40 large T antigen (Tag) performs a similar molecular role as *Cdkn2a* loss [21] In both the wild type and MexTAg model, asbestos-induced MM was not associated with reduced expression of other genes commonly down-regulated in human MM [21].

To date no common oncogenic driver has been identified in MM, although amplification of the C-MYC locus in MM has been reported to occur in MM human tumour cell lines and may contribute to a malignant phenotype [52]. In the current study, increased expression of c-Myc was observed, however no other recognized oncogenes were identified in significantly amplified regions across the samples. It is important to acknowledge that, despite these particular CNVs being previously reported in human MM, the selection pressure of culture conditions may account for some CNV in this study. Notably, however, there was no correlation between the number of CNVs and time in culture in this study.

Carcinogen-induced cancers such as melanoma and lung cancer typically show a high rate of mutation when compared to spontaneous cancers [6–8]. Given that MM is also a carcinogen induced tumour, the average somatic mutation rate in human MM is lower than expected [7, 8]. Here we show that the CBA and C57BL/6 strains of the wild-type murine model of MM display a comparable mutation rate to human MM. Interestingly, the BALB/c strain showed a wider range of mutation rates between the samples. Previously, germline mutations in a key DNA damage sensor gene, *Prkdc*, have been reported in some colonies of BALB/c mice [53]. These mutations were also present in the BALB/c mice used to generate the cell lines used in this study, possibly resulting in the current and previously reported genomic instability of tumour cell lines from this strain [5] and may account for the higher rate of spontaneous mutation when exposed to asbestos.

Patterns of mutations can be used to identify mutational signatures which represent the underlying process of DNA damage, (for example tobacco smoking induces a mutational pattern that exhibits transcriptional strand bias for C > A mutations and CC > AA dinucleotide substitutions) [6]. To date no asbestos-related mutation signature has been identified. The current exome sequencing study was underpowered to perform a full mutational signature analysis but did show a high rate of C > T and G > A mutations, consistent with previous studies in humans [7, 8]. Whole genome sequencing of a larger cohort of samples will be required to fully answer this question in both humans and mice.

Given the genetically independent nature of MM tumours and the low likelihood of the same gene being mutated at different positions in more than one sample, the presence of different mutations in *Nkd1* in two BALB/c samples and *Cacna2d2* in samples from two different strains is informative. The mutation of *Nkd1* may implicate the role of Wnt signaling pathway regulation in the BALB/c strain. Genes involved in the Jak-STAT and MAPK signaling pathways were also altered in the murine model. These Wnt, Jak-STAT and MAPK pathways have been identified as being altered in many cancers, including MM. In humans, *CACNA2D2* is located 1.9 Mb upstream from *BAP1* in the region 3p21. However, in the mouse genome, the orthologs reside on different chromosomes. The importance of this gene in MM in undetermined, though it has been linked to tumorigenesis in prostate cancer [46] and loss of expression has been demonstrated in non-small cell lung cancer [54].

## Conclusion

Our study is the first to molecularly characterise three extensively utilized strains of a wild-type model of asbestos-induced MM and catalogue the somatic mutations and copy number variations they contain. Previous studies have reported on small numbers of wild-type murine MM tumours within larger studies focusing on transgenic models [5, 19, 20, 22, 24]. We confirm that asbestos exposure in wild-type mice causes MM tumour development through homozygous loss of *Cdkn2a* leading to loss of expression of p16 and that this is an initiating event of MM tumorigenesis, a loss that is commonly identified in over 70% of human MM tumours. We find that wild-type asbestos-induced murine MM harbors a similar mutation rate to human MM and that copy number deletions dominate the mutational landscape, particularly within similar pathways as human MM. This study provides a foundation for further research into the wild-type murine MM model at the translational level. Our findings provide support for the use of a wild-type murine model of asbestos-induced malignant mesothelioma as an invaluable tool for the study of the molecular basis of human MM, as well as provide the means to study early events in MM development post asbestos exposure, the search for novel therapies based on identification of druggable molecular targets, carcinogen signature evaluation and biomarker studies.

## Additional files

Additional file 1: Table S1. Sequencing metrics and summary of somatic mutations and copy number variations. (DOCX 111 kb)

Additional file 2: Table S2. Genes of interest previously reported in MM and primer sequences used to detect expression of the targets in murine messenger RNA. (DOCX 85 kb)

Additional file 3: Table S3. Copy number variations determined as significant across all 15 tumour cell line samples (q-value threshold <0.1). (DOCX 141 kb)

## Abbreviations

aCGH: Array comparative genomic hybridisation
Bap1: BRCA1 associated protein 1
Cdkn2a: Cyclin dependent kinase inhibitor 2A
CIGAR: Concise idiosyncratic gapped alignment report
CNV: Copy number variation
DNA: Deoxyribonucleic acid
GATK: Genome Analysis Toolkit
GISTIC: Genetic identification of significant targets in cancer
IGV: Integrative Genomics Viewer
Indel: Short insertion or deletion
KEGG: Kyoto Encyclopedia of Genes and Genomes
Mb: Million bases
MD: Match/mismatch
MM: Mesothelioma
mRNA: Messenger RNA
Nf2: Neurofibromin 2
RNA: Ribonucleic acid
RT-PCR: Reverse transcription polymerase chain reaction
SNV: Single nucleotide variation
Trp53: Tumour protein 53
UV: Ultraviolet
-x: -fold coverage
