# Supplementary material for: Whole exome sequencing of an asbestos-induced wild-type murine model of malignant mesothelioma
Source: BMC Cancer. 2017 Jun 2;17:396. doi: 10.1186/s12885-017-3382-6 (PMC5455120; doi:10.1186/s12885-017-3382-6)
Supplement: Supplementary file 1 — Sequencing metrics and summary of somatic mutations and copy number variations. (DOCX 111 kb) [file 12885_2017_3382_MOESM1_ESM.docx]

| **Supplementary table S1: Sequencing metrics and summary of somatic mutations and copy number variations** | | | | | | | | | | |
| --- | --- | --- | --- | --- | --- | --- | --- | --- | --- | --- |
| **Sample** | **Strain** | **Passage** | **Sample Type** | **Average coverage (x)** | **Coverage of 80% of target bases (x)** | **Total mutations** | **Missense mutations** | **Indels** | **Mutation rate (per Mb)** | **No. total CNV events** |
| AB1 | BALB/c | 18 | Tumour | 30.65 | 11 | 438 | 327 | 2 | 8.9 | 130 |
| AB12 | BALB/c | 11 | Tumour | 25.83 | 6 | 70 | 56 | 1 | 1.4 | 1630 |
| AB13 | BALB/c | 14 | Tumour | 24.74 | 6 | 149 | 123 | 0 | 3.0 | 208 |
| AB22 | BALB/c | 8 | Tumour | 35.99 | 9 | 242 | 177 | 0 | 4.9 | 208 |
| AC16 | CBA | 24 | Tumour | 36.3 | 10 | 30 | 19 | 1 | 0.6 | 1726 |
| AC24 | CBA | 31 | Tumour | 54.38 | 17 | 46 | 27 | 2 | 1.0 | 347 |
| AC28 | CBA | 13 | Tumour | 51.62 | 16 | 45 | 35 | 1 | 0.9 | 349 |
| AC29 | CBA | 31 | Tumour | 46.02 | 14 | 22 | 16 | 3 | 0.5 | 264 |
| AC31 | CBA | 8 | Tumour | 62.66 | 20 | 22 | 12 | 0 | 0.4 | 352 |
| AE3 | C57BL/6 | 13 | Tumour | 53.92 | 17 | 28 | 19 | 0 | 0.6 | 139 |
| AE16 | C57BL/6 | 14 | Tumour | 43.17 | 13 | 33 | 23 | 1 | 0.7 | 249 |
| AE17 | C57BL/6 | 13 | Tumour | 34.94 | 17 | 56 | 36 | 0 | 1.1 | 1479 |
| AE19 | C57BL/6 | 17 | Tumour | 58.38 | 19 | 23 | 14 | 0 | 0.5 | 110 |
| BM109 | C57BL/6 | 14 | Tumour | 51.26 | 17 | 33 | 27 | 1 | 0.7 | 1571 |
| BM163 | C57BL/6 | 20 | Tumour | 40.42 | 13 | 34 | 28 | 0 | 0.7 | 121 |
| BALB/c |  |  | Wild-type | 30 | 11 | - | - | - | - |  |
| CBA |  |  | Wild-type | 32.06 | 10 | - | - | - | - |  |
| C57BL/6 |  |  | Wild-type | 38.87 | 13 | - | - | - | - |  |
