# Supplementary material for: Whole exome sequencing of an asbestos-induced wild-type murine model of malignant mesothelioma
Source: BMC Cancer. 2017 Jun 2;17:396. doi: 10.1186/s12885-017-3382-6 (PMC5455120; doi:10.1186/s12885-017-3382-6)
Supplement: Supplementary file 2 — Genes of interest previously reported in MM and primer sequences used to detect expression of the targets in murine messenger RNA. (DOCX 85 kb) [file 12885_2017_3382_MOESM2_ESM.docx]

| **Supplementary table S2: Genes of interest previously reported in MM, and primer sequences used to detect expression of the targets in murine messenger RNA** | | | |
| --- | --- | --- | --- |
| **Gene** | **Forward primer sequence** | **Reverse primer sequence** | **Previously reported in MM** |
| *Cdkn2a* | CGTACCCCGATTCAGGTGAT | CCAGCGGAACGCAAATATCG | ^1^ |
| *Nf2* | TAAAAAGCCTCAAGCCCAAGG | CCAAACAAGCCAGCCCTCTA | ^1,2^ |
| *Bap1* | TGGCTCAGGAAGGAATGCTG | CAGAGGTTCTCACTGGCGTT | ^1-3^ |
| *Cul1* | GTTTGCTTGAGGAACAGCGG | ACATGCGGCCCAAATCTTCA | ^1^ |
| *Trp53* | GGAAGTCCTTTGCCCTGAACT | GTCTTCAGGTAGCTGGAGTGAG | ^2^ |
| *Setd2* | CCCAAAGACAGCAATGGCAC | TCGTTCAGCTACTGTGCTCTC | ^2^ |
| *Lats2* | CAAGCAGACCTCCCCAGGAAAG | GGCTCCAGGGAAGAGAAAGTC | ^4^ |
| *Myc-a* | CTCTGGAGTGAGAGGGGCTT | TGGCTGTCTGCGGGGTTTC |  |
| *Myc-b* | GTTGGAAACCCCGACAGCC | CGACCGCAACATAGGATGGA |  |
