# Supplementary material for: Whole exome sequencing of an asbestos-induced wild-type murine model of malignant mesothelioma
Source: BMC Cancer. 2017 Jun 2;17:396. doi: 10.1186/s12885-017-3382-6 (PMC5455120; doi:10.1186/s12885-017-3382-6)
Supplement: Supplementary file 3 — Copy number variations determined as significant across all 15 tumour cell line samples (q-value threshold <0.1). (DOCX 141 kb) [file 12885_2017_3382_MOESM3_ESM.docx]

| **Supplementary table S3: Copy number variations determined as significant across all 15 tumour cell line samples (q-value threshold <0.1)** | | | | | |
| --- | --- | --- | --- | --- | --- |
| **Locus** | **Type** | **Region** | **Length (Mbp)** | **Genes** | **Residual q-value** |
| 5qA2 | Amplification | chr5:14811483-15934945 | 1.1 | *Cacna2d1, Pclo, Gm17019, Speer8-ps1, 4930519H02Rik, Speer4cos, Speer4d, Gm9758, 4930572O03Rik, Speer4e, Speer4c, Gm10354, Gm21190, LOC105242399* | 8.09x10^-3^ |
| 4qE2 | Amplification | chr4:155659590-155957427 | 0.3 | *Dvl1, Ccnl2, Aurkaip1, Mrpl20, Ssu72, Atad3aos, Cpsf3l, Mxra8, Mib2, Cptp, Tas1r3, Atad3a, Ube2j2, Acap3, B930041F14Rik, Vwa1, Tmem88b, Tmem240, Pusl1, Gm5801* | 1.50x10^-2^ |
| 4qC4 | Deletion | chr4:88721449-90361061 | 1.6 | *Cdkn2a, Cdkn2b, Ifna1, Ifna11, Ifna4, Ifna5, Ifna6, Ifna7, Mtap, Ifne, Zfp352, Dmrta1, Ifnz, Gm13271, Gm13283, Gm13289, Gm13272, Gm13276, Gm13277, Gm13278, Gm13275, Gm13279, Gm13285, Gm13288, Mir31, Gm12603, Gm13286, Gm12610, LOC106557447* | 5.07x10^-17^ |
| 7qA1 | Deletion | chr7:7324675-10162881 | 2.8 | *Clcn4, Vmn2r37, Vmn2r30, Vmn2r42, Vmn2r32, 2810047C21Rik1, Vmn2r43, Vmn2r38, Vmn2r44, Vmn2r50, Vmn2r39, Vmn2r33, Vmn2r35, Vmn2r48, Vmn2r49, Vmn2r31, Vmn2r34, Vmn2r36, Vmn2r40, Vmn2r45, Vmn2r41, Vmn2r47, Vmn2r46, Vmn2r51, Mir5620* | 2.7x10^-7^ |
| 13qB3 | Deletion | chr13:65297239-66901259 | 1.6 | *2410141K09Rik, Gm10324* | 6.41x10^-6^ |
| 12qC1 | Deletion | chr12:54975051-55297702 | 0.3 | *Srp54a, 2700097O09Rik, Srp54b, Srp54c, 1700047I17Rik2* | 9.95x10^-5^ |
| 14qA3 | Deletion | chr14:18526031-19816095 | 1.3 | *Nid2, Ube2e2, Gm5458* | 1.36x10^-3^ |
| 9qE3.1 | Deletion | chr9:88642936-89998653 | 1.4 | *Bcl2a1a, Bcl2a1b, Bcl2a1d, Rasgrf1, Tmed3, 4930524O08Rik, Mthfs, AF529169, 9330159M07Rik, Ankrd34c, Mir184, Trim43c, Trim43b, Mthfsl* | 2.66x10^-2^ |
| 2qH4 | Deletion | chr2:174779622- 178302617 | 3.6 | *Gm14288, C330013J21Rik, Gm14325, Zfp931, Gm14403, Gm14322, Gm6710, Etohi1, Gm14405, Gm14430, Gm14420, Gm14393, Gm14391, Gm14327, Gm14326, Gm8898, OTTMUSG00000016609, Gm14434, 0610010B08Rik, Gm14295, Gm14308, Gm14305, Gm14436, Gm14431, Gm14440, Gm8923, 2210418O10Rik, Gm14296* | 3.11x10^-2^ |
| 16qC1.3 | Deletion | chr16:59554031-75873550 | 16.3 | *Epha3, Epha6, Htr1f, Pou1f1, Pros1, Robo1, D16Ertd519e, Arl6, 4930453N24Rik, Arl13b, Stx19, Chmp2b, 4931420L22Rik, Zfp654, Vgll3, Gbe1, 4930547E14Rik, 1700010K23Rik, 4930428D20Rik, 4930567J20Rik, Cggbp1, Nsun3, Hspa13, Csnka2ip, Speer2, Rbm11, Cadm2, Robo2, 8030451O07Rik, Lipi, Mir691* | 3.53x10^-2^ |
| 6qF3 | Deletion | chr6:129582978-133735492 | 4.1 | *Klra1, Klra10, Klra12, Klra13-ps, Klra2, Klra3, Klra4, Klra5, Klra6, Klra7, Klra8, Klra9, Klrc1, Klrc2, Klrd1, Prh1, Klrk1, Klra15, Ybx3, Tas2r105, Klrc3, Magohb, 5430401F13Rik, Klra23, Prp2, Klra21, Klra22, Klra18, Klra19, Tas2r116, Gm4736, Klra17, Gm156, 5530400C23Rik, Styk1, Klri2, Tas2r136, Tas2r117, Tas2r123, Tas2r115, A630073D07Rik, Smim10l1, Prpmp5, Prb1, Tas2r102, Tas2r104, Tas2r106, Tas2r107, Tas2r109, Tas2r110, Tas2r113, Tas2r114, Tas2r120, Tas2r121, Tas2r124, Tas2r125, Tas2r129, Tas2r130, Tas2r131, Tas2r140, Klri1, Gm5885, Tas2r122, Klra14-ps, Gm8882, Tas2r103, Mir680-1* | 4.50x10^-2^ |
| 1qF | Deletion | chr1:139478359-140609542 | 1.1 | *Cfh, F13b, Cfhr1, Gm4788, Cfhr2* | 5.06x10^-2^ |
| 10qC1 | Deletion | chr10:81507927-82649863 | 1.1 | *Gna11, Aes, Tle2, Sirt6, Ankrd24, Zfp433, Tle6, AU041133, Zfp938, BC025920, Zfp781, Zfp873, Gm1553, 1190007I07Rik, Gm10778* | 5.34x10^-2^ |
| 5qE3 | Deletion | chr5:93270777-96079904 | 2.8 | *Cxcl13, AA792892, D5Ertd577e, A430089I19Rik, C87414, BC061212, Gm16367, Gm6367, E330014E10Rik, Gm3139, Gm16513, Gm3259, Gm3286* | 5.67x10^-2^ |
